# The Mini-BESTest - a clinically reproducible tool for balance evaluations in mild to moderate Parkinson’s disease?

**DOI:** 10.1186/s12883-014-0235-7

**Published:** 2014-12-12

**Authors:** Niklas Löfgren, Emma Lenholm, David Conradsson, Agneta Ståhle, Erika Franzén

**Affiliations:** Karolinska Institutet, Department of Neurobiology, Care Sciences and Society, Division of Physiotherapy, 23100, SE-14183 Huddinge, Sweden; Department of Physical Therapy, Karolinska University Hospital, Stockholm, Sweden

**Keywords:** Reliability, Measurement error, Psychometric, Balance, Balance evaluation systems test, Test-retest, Inter-rater, Smallest real difference

## Abstract

**Background:**

The Mini-BESTest is a clinical balance test that has shown a high sensitivity in detecting balance impairments in elderly with Parkinson's disease (PD). However, its reproducibility between different raters and between test occasions has yet to be investigated in a clinical context. Moreover, no one has investigated the reproducibility of the Mini-BESTest's subcomponents (i.e. anticipatory postural adjustments; postural responses; sensory orientation and dynamic gait).

We aimed to investigate the inter-rater and test-retest reproducibility (reliability as well as agreement) of the Mini-BESTest, as well as its subcomponents, in elderly with mild to moderate PD, performed under conditions assimilating clinical practice.

**Method:**

This was an observational measurement study with a test-retest design.

Twenty-seven individuals with idiopathic PD (66 - 80 years, mean age: 73; Hoehn & Yahr: 2-3; 1-15 years since diagnosis) were included. Two test administrators, having different experiences with the Mini-BESTest, administered the test individually, in separate rooms in a hospital setting. For the test-retest assessment, all participants returned 7 days after the first test session to perform the Mini-BESTest under similar conditions. Intra-class correlation coefficients (ICC_2.1_), standard error of measurement (SEM_agreement_), and smallest real difference (SRD) were analyzed.

**Results:**

The Mini-BESTest showed good reliability for both inter-rater and test-retest reproducibility (ICC = 0.72 and 0.80). Regarding agreement, the measurement error (SRD) was found to be 4.1 points (accounting for 15% of the maximal total score) for inter-rater reproducibility and 3.4 points (12% of the maximal total score) for test-retest reproducibility. The investigation of the Mini-BESTest's subcomponents showed a similar pattern for both inter-rater and test-retest reproducibility, where postural responses had the largest proportional measurement error, and sensory orientation showed the highest agreement.

**Conclusions:**

Our findings indicate that the Mini-BESTest is able to distinguish between individuals with mild to moderate PD; however, when used in clinical balance assessments, the large measurement error needs to be accounted for.

## Background

Parkinson’s disease (PD), with a prevalence of more than 4 million people worldwide [1] and 22,000 in Sweden [2], is the second most common neurodegenerative disease. The incidence of PD, most common after the age of 60 years [3], rises with age and is expected to grow rapidly in the coming years [1]. As the disease progresses, impaired balance control becomes one of the main features, interfering with physical independence [4] and quality of life [5,6]. Out of the four cardinal symptoms in PD (tremor, bradykinesia, rigidity and postural instability), all but tremor are related to impaired balance control [4,7]. Balance control in PD, being negatively affected from the early stages of the disease, includes impairments of postural responses as well as with turning and gait, and is also related to an increased risk of falling [4]. It is therefore vital for a balance test to identify the array of problems that may occur in an individual with PD.

In order to help clinicians identify the specific balance problems, the Balance Evaluation Systems Test (BESTest) [8] was developed, addressing a variety of components influencing balance control. However, being very comprehensive, it has been regarded as too time consuming. Instead, this led to the development of the shortened, more clinically applicable version; the Mini-BESTest [9]. This test addresses subcomponents of balance control, such as anticipatory postural adjustments, postural responses, sensory orientation and dynamic gait, and has been found to be sensitive in disclosing balance impairments among individuals with PD [10].

Reproducibility concerns the degree to which repeated measurements in study objects provide similar results, and can be divided into parameters of reliability or agreement [11]. Whereas reliability measures aim to distinguish study objects from each other despite measurement errors, measures of agreement assesses the absolute measurement error of a test (i.e., the exact measurement error presented in the same units as the investigated item) [11,12]. When investigating the reproducibility of a clinical test, there are various aspects to take into consideration. Sources of error for a test may depend upon the test administrator, the tested subject, the instrumentation and/or biological variability, as well as of the circumstances under which the test takes place [11,13]. To gain fully accountable information regarding the reliability of a test, it is therefore important to take all possible sources of error into account. However, the prevailing approach for reproducibility investigations tend to be to use one person to act as a test administrator, being responsible for test instructions and the safety of the patient, and one or more other persons that observe and rate the test performance (by using the test instructions regarding how to grade the test performance). Although such an observational approach investigates the reproducibility of the test itself (by testing the actual grading scale), it lacks ecological validity because such conditions do not assimilate clinical practice. Indeed, the assessment of balance performance in clinical practice is a rather complex situation, with the clinician generally being responsible for giving the patients clear and concise instructions, as well as for the adequate interpretation (rating) of the test performance and the safety of the patient. Such a situation is also dependent on the relation between the clinician and the patient, that is, whether or not the patient trusts the clinician enough to perform challenging tasks adequately.

Previous studies have used the observational approach of investigating inter-rater reproducibility of the Mini-BESTest [8,14-18], whereas others have used video recordings to rate the test retrospectively [19-21]. Such approaches differ from clinical test situations, making it difficult to generalize the results derived from these studies to clinical practice. Moreover, at times different physical therapists (with varied experience) may assess the balance abilities of a single patient before and after a rehabilitation period or at different stages in the health care system. Therefore, it may be important also to investigate the reproducibility of clinical tests that are performed by clinicians with different experience.

Recent studies have found the Mini-BESTest to be highly reproducible [14,15,18,22], but only one study focused on individuals with PD exclusively [14]. In addition, the majority of these studies have reported their results only in form of the reliability parameter intra-class correlation (ICC). From a clinical perspective, this may be problematic, as the ICC score is a relative measure of reproducibility, thereby depending on the variance between subjects (i.e., the ICC score will be higher if there is much variability between subjects even if the variability between tests is high, and vice versa) [13,23]. Therefore, the ICC score preferably should be complemented with measures of agreement (i.e., the exact number of points that are likely to reflect the measurement error), such as the standard error of measurement (SEM) [12] and the smallest real difference (SRD) [24]. The SEM estimates an exact score that reflects the within subject error variance, and can be presented as SEM_agreement_ (including systematic differences) or SEM_consistency_ (excluding systematic differences) [11]. When the SEM is known, the SRD (reflecting the exact measurement error in a single individual) can be calculated [24].

Finally, as the reproducibility of the assessment may vary between different areas of balance control, it might also be of significance to investigate the reproducibility of each of the Mini-BESTest’s subcomponents. Our aim was therefore to investigate, in a clinical context, the inter-rater and test-retest reproducibility of the Mini-BESTest and its subcomponents in elderly with mild-to-moderate PD.

## Method

This study was designed as an observational measurement study with a test-retest design.

The Regional Board of Ethics in Stockholm (Dnr:2009/819-32, 2010/1472-32 and 2012/1829-32) provided their ethical approval of the study.

### Participants

The participants were recruited from a balance intervention study (BETA-PD trial [25]) and provided their written informed consent to participate. However, none of the participants took part in any kind of physical intervention during the time of this study.

Inclusion criteria were a clinical diagnosis of “idiopathic” PD [26], Hoehn and Yahr stages 2 and 3, and ≥ 60 years of age. Participants were excluded if they had a diagnosis of other existing neurological disorders and/or medical conditions affecting balance control.

Twenty-seven elderly (9 females; mean age 73; SD 4.1) with mild to moderate PD (Hoehn & Yahr, stage 2, n = 16; stage 3, n = 11) participated in this study; see Table 1. All participants had a Mini Mental State Examination (MMSE) [27] score of at least 24 points (indicating adequate cognitive function to occur in this sample) and were tested during the on-phase with regards to their medication scheme. Twenty-four of the participants reported self-perceived balance impairments, eight had experienced at least one fall during the past 12 months, 12 were afraid of falling (answering “yes” or “no” to a direct question), and one used a walking aid (cane).

**Table 1:** **Participant demographics (n = 27)**

	**Mean**	**SD**	**Range**
Age (years)	73	4	66-80
Height (cm)	171	10	145-183
Weight (kg)	74	16	46-101
Body mass index (weight/length^2^)	25	3	18–31
Time since diagnosis (years)	6.2	4	1-15
UPDRS motor score	35	11	11-63

UPDRS = Unified Parkinson’s disease rating scale.

### Outcome measures/assessment

The Mini-BESTest (also presented in Table 2) is a balance test consisting of 14 items, including tasks divided into four subcomponents: anticipatory postural adjustments, postural responses, sensory orientation, and dynamic gait. Items are scored from 0 (unable or requiring help) to 2 (normal) on an ordinal scale with the maximal total score of 28 points. The items; single limb stance and compensatory stepping correction (lateral), were assessed on both the right and left sides. However, only the score of the worst side was used to calculate the total score [9,28].

**Table 2:** **Summary of the subcomponents and the items of the Mini-BESTest** ^**1**^

***Anticipatory postural adjustments*** ^***2***^	***Sensory orientation*** ^***2***^
- Sit to stand	- Stance on firm surface; eyes open
- Rise to toes	- Stance on foam surface; eyes closed
- Stand on one leg (right and left)^4^	- Stance on incline surface, eyes closed
***Postural responses*** ^***2***^	***Dynamic gait*** ^***3***^
- Compensatory stepping correction; forward	- Change in gait speed
- Compensatory stepping correction; backward	- Walk with head turns; horizontal
- Compensatory stepping correction; lateral (right and left)^4^	- Walk with pivot turns
	- Step over obstacles
	- Timed up & go with dual-task

^1^Maximal total score = 28 points, ^2^Maximal subcomponent score = 6 points, ^3^Maximal subcomponent score = 10 points, ^4^Only the score of the worst side was used to calculate the total score [9,28].

### Procedure

To investigate inter-rater reproducibility, two physical therapists with different experiences of the Mini-BESTest administered and rated the test performance on the same day at Karolinska University Hospital, Stockholm. The more-experienced rater (rater A) had administered and rated the Mini-BESTest more than 100 times, whereas the less-experienced rater (rater B) had administered the test approximately ten times before this study started. To synchronize their assessment of the Mini-BESTest, the two raters met prior to the study on two occasions to discuss the principles of the test, and to practice its administration and rating. However, during and after the test sessions the raters were blinded to each other’s ratings.

Participants were briefly interviewed, using a standardized protocol, regarding current health status including years since diagnosis. Disease severity was measured with the Unified Parkinson’s Disease Rating Scale (UPDRS) [29], and cognitive function was assessed with the MMSE [27]. Subsequently, the participants performed the Mini-BESTest with each of the two test administrators, who were situated in separate rooms. Randomization decided which administrator to start with. The test procedure took approximately one hour to complete.

For test-retest reproducibility, the more experienced rater (rater A) reassessed the participants seven days later. At the second test session, rater A performed a brief interview, including questions regarding pain, medication, activity, falls, and other possible incidents that might have influenced their balance performance since the previous session. Following this, the participants performed the Mini-BESTest at the same location and time of the day as they had performed the test the previous week.

### Data analysis

Statistical analyses were performed with SPSS (version 22, SPSS Inc., Chicago, IL). Cronbach’s alpha was used to assess the internal consistency, where values of at least 0.7 were considered acceptable [30]. Reliability was investigated by means of ICC_2.1_ where one-way repeated measures analysis of variance (ANOVA) were used to calculate agreement between raters (inter-rater reproducibility) and test sessions (test-retest reproducibility), regarding the total score of the Mini-BESTest as well as its subcomponents. To categorize the level of ICC agreement, we used Altman’s classification: < 0.20 = poor; 0.21–0.40 = fair, 0.41–0.60 = moderate, 0.61–0.80 = good, 0.81–1.0 = very good [31].

For parameters of agreement, first SEM_agreement_ was calculated as follows: SEM = √within subject error variance [32]. Following this, the SRD was calculated with a 95% Confidence Interval (CI), resulting in the following formula: SRD = 1.96 × √2 × SEM [24]. Moreover, to evaluate the proportion of the measurement error, we calculated the SRD% by dividing the SRD with the maximal total score of the Mini-BESTest (28 points). Similarly, the SRD of each subcomponent was divided with its maximal total score (6 or 10 points). To analyze systematic changes of the mean between testers and test sessions, we used Bland-Altman plots [33].

## Results

All participants completed both test sessions. None of the participants changed their PD medication between the test sessions, nor did they report that any forms of adverse events or change of health status had occurred.

### Inter-rater reproducibility

**Figure 1:**
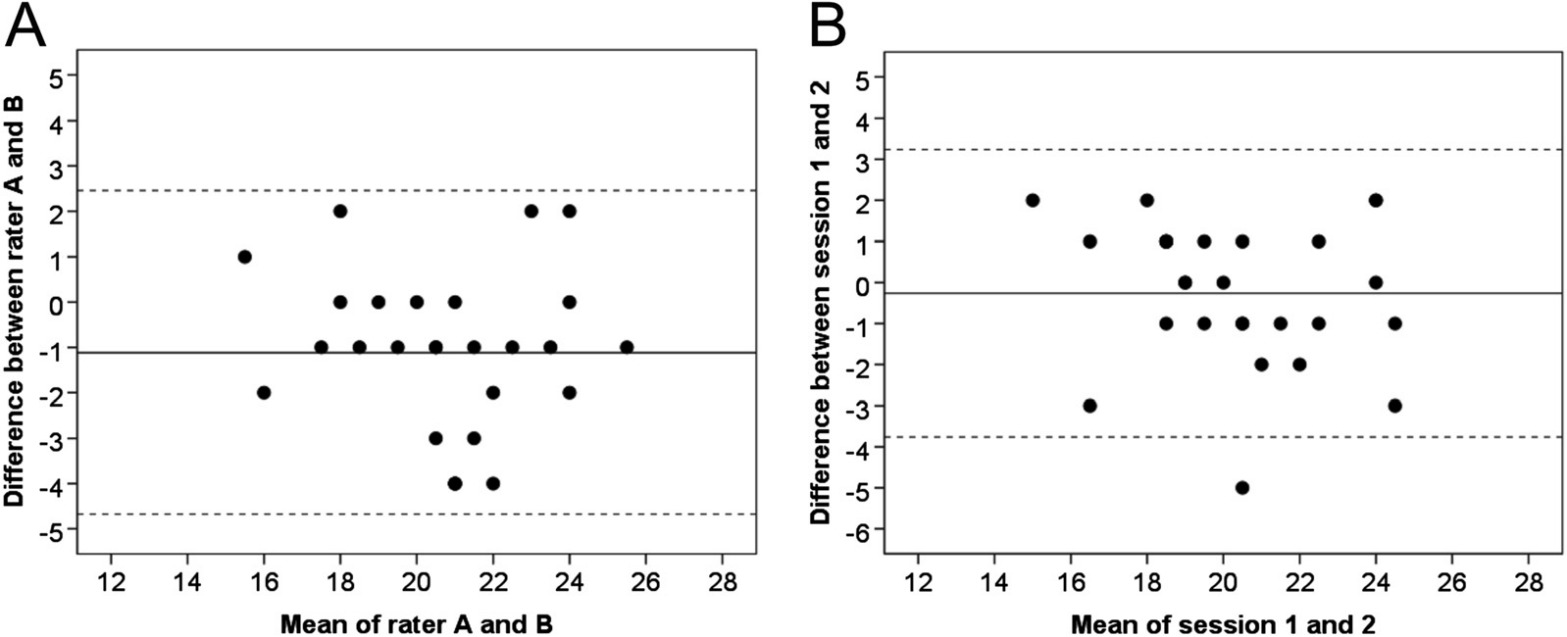
**Bland & Altman graphs presenting the Mini-BESTest for inter-rater (A) and test-retest (B) reliability. (A)** The difference between rater **A** and **B** is plotted against the mean of rater A and B. **(B)** The difference between test session 1 and 2 is plotted against the mean of the two sessions. The solid line represents the mean difference between the two tests and the dotted line two standard deviations (limits of agreement).

The participants’ mean total score of the Mini-BESTest was found to be 20.2 points when assessed by rater A and 21.3 points when assessed by rater B (Table 3). Regarding inter-rater reproducibility, the Mini-BESTest showed good reliability (ICC = 0.72) and acceptable internal consistency (Cronbach’s alpha = 0.87). In addition, our findings on agreement showed the SEM to be 1.5 points, whereas the SRD score revealed a measurement error of 4.1 points (15% of the maximal total score). The Bland and Altman graph (Figure 1A) illustrates the occurrence of a systematic difference, showing that rater B scored the participants higher than did rater A (p = 0.003). However no heteroscedasticity was observed. Our findings on the subcomponents of the Mini-BESTest showed the measurement error to be highest for the postural responses (38% of the maximal subcomponent score). Conversely, the measurement error was lowest for sensory orientation (17% of the maximal subcomponent score).

**Table 3:** **Inter-rater and test-retest reproducibility of the Mini-BESTest and its subcomponents**

	**Mean**	**SD**	**Range**	**Mean**	**SD**	**Range**	***P*** **-value**	**ICC**_**2.1**_	**95% CI**	**Cronbach’s alpha**	**SEM**_**agreement**_	**SRD**	**SRD%**
***Inter-rater reproducibility***	**Rater A**			**Rater B**									
Mini-BESTest, total score	20.2	2.6	15-25	21.3	2.7	15-26	*0.003*	0.72	0.37-0.87	0.87	1.5	4.1	14.6^1^
Anticipatory postural adjustments	3.6	1.2	1-6	4.1	1.0	2-6	*0.007*	0.65	0.31-0.83	0.83	0.7	1.9	31.7^2^
Postural responses	4.2	1.0	3-6	3.7	1.1	1-6	*0.043*	0.43	0.09-0.69	0.63	0.8	2.3	38.3^2^
Sensory orientation	5.7	0.6	4-6	5.9	0.5	4-6	0.265	0.54	0.22-0.76	0.70	0.4	1.0	16.7^2^
Dynamic gait	6.7	1.4	5-10	7.7	1.1	5-9	*0.000*	0.48	0.24-0.75	0.75	1.1	2.9	29.0^2^
***Test-retest reproducibility***	**Session 1**			**Session 2**									
Mini-BESTest, total score	20.2	2.6	15-25	20.5	2.9	14-26	0.447	0.80	0.60-0.90	0.88	1.2	3.4	12.1^1^
Anticipatory postural adjustments	3.6	1.2	1-6	3.7	1.1	2-6	0.602	0.79	0.60-0.90	0.88	0.5	1.4	23.3^2^
Postural responses	4.2	1.0	3-6	4.4	1.1	3-6	0.110	0.70	0.45-0.85	0.83	0.6	1.6	26.7^2^
Sensory orientation	5.7	0.6	4-6	5.9	0.4	5-6	0.185	0.54	0.33-0.81	0.77	0.3	0.8	13.3^2^
Dynamic gait	6.7	1.4	5-10	6.5	1.7	4-10	0.363	0.78	0.57-0.89	0.87	0.7	2.0	20.0^2^

SD = Standard Deviation, ICC = Intra Class Correlation, CI = Confidence Interval, SEM = Standard Error of Measurement (√within subjects error variance), SRD = Smallest Real Difference (1.96 × √2 × SEM), SRD% = ^1^(SRD/maximal total score) × 100, ^2^(SRD/maximal subcomponent score) × 100.

### Test-retest reproducibility

The participants’ mean total score of the Mini-BESTest was found to be 20.2 points for test session one and 20.5 for test session two (Table 3). The Mini-BESTest showed good reliability (ICC = 0.80) and acceptable internal consistency (Cronbach’s alpha = 0.88). Our findings on agreement showed a measurement error of 3.4 points (12% of the maximal total score). The Bland-Altman graph (Figure 1B) showed that, apart from one outlier, no heteroscedasticity was observed. Regarding the agreement of the Mini-BESTest’s subcomponents, we found the measurement error to be highest for the postural responses (27% of the maximal subcomponent score). Conversely, the measurement error was lowest for sensory orientation (13% of the maximal subcomponent score).

## Discussion

This is the first study to use a methodology similar to clinical practice to investigate the reproducibility of the Mini-BESTest, as well as its subcomponents, in PD. We found good reliability [31] (ICC > 0.70), as well as acceptable internal consistency [30] (Cronbach’s alpha = 0.87 and 0.88, respectively), for both inter-rater and test-retest reproducibility. However, the agreement was considered low [34] with the measurement error accounting for, respectively, 15% (4.1 points) and 12% (3.4 points) of the maximal total score.

Seemingly, the reliability of both inter-rater and test-retest reproducibility in this study was good. Nevertheless, these results may seem low compared to prior studies of the Mini-BESTest, with inter-rater ICC scores ranging from 0.91 to 0.99 [14,15,18,22] and where test-retest scores have ranged from 0.88 to 0.97 [14,15,18,22]. However, it is difficult to compare directly our results on inter-rater reproducibility with previous studies because either our methodology (with two independent raters) or the participants differs. Indeed, whereas our methodology is comparable to the study of Tsang et al., [22] they investigated individuals with chronic stroke. Considering the prevalence of fluctuations of both motor [35] and non-motor [36] symptoms in PD, this makes a direct comparison rather faulty. Leddy et al. [14] on the other hand, studying exclusively elderly with PD, used only one person to administrate the test, whereas three observers rated the test performance. We consider this to be a method that tests the reproducibility of the rating scale of the Mini-BESTest, rather than its reproducibility from a clinical perspective. Moreover, because ICC values are influenced by variability [23], it is possible that the reasonably low degree of variability in this study (including only participants at Hoehn & Yahr stages 2 and 3, and with the range of Mini-BESTest scores accounting for approximately 40% of the total test score) affected the ICC values negatively.

Hitherto, no previous studies have reported the inter-rater or test-retest agreement of the Mini-BESTest in PD. However, in individuals with mild-to-moderate stroke, Tsang et al. [22] found the measurement error to be 3 points (11% of the maximal total score) with a test-retest design, which was similar to what we found. Moreover, our results of test-retest agreement are also comparable to those found by Steffen et al. [37] on the Bergs balance scale in PD. Whereas they found the measurement error to be 5 points out of 56 (9% of the maximal test score), our results of 3.4 points out of 28 (12% of the maximal test score) are just slightly higher. Seemingly, the aforementioned studies (well-designed by appearance and performed in different contexts), similar to ours, have found the measurement error to account for approximately 10% of the total score. Measurement errors of that size, requiring changes of a magnitude that is rare to achieve [38-40], are bound to make it difficult for clinicians to rely on their results with confidence (thereby limiting the instruments clinical value). However, it might be relevant to highlight that these results have been calculated with the prevailing and rather strict formula containing 95% confidence [24,32] (meaning that one can be 95% certain that the results are correct). One might consider if it could be of higher clinical value to calculate the measurement error with 80-90% confidence instead [41], something likely to result in a more manageable measurement error that can be relied upon with 80-90% certainty.

The analysis of the subcomponents of the Mini-BESTest showed that sensory orientation (consisting of only stationary exercises) had the highest agreement, both regarding inter-rater and test-retest reproducibility. Accordingly, the more dynamic subcomponent of postural responses had the lowest agreement. These results were in accordance with those found by Tsang et al. [22], where items associated with postural responses showed the lowest agreement. This is not surprising because the reactive postural responses subcomponent includes asking persons, possibly frightened of falling, to lean their bodyweight into the hands of the test administrator, who also needs to be consistent regarding how far to lean the persons before suddenly releasing the support. Such a task seems likely to be more challenging than simply to assess the time a person is able to stand on a foam surface with his or her eyes closed. One might argue that these kinds of challenging, nevertheless important, items may be likely to require skilled and well-trained raters.

At inter-rater reproducibility, a systematic bias occurred between the raters, revealing the less experienced rater to score the subjects higher (Figure 1). However, the difference in results between raters (SRD = 4.1) was only marginally larger than between a single rater at two separate occasions (SRD = 3.4). This may indicate that both administrators had a similar understanding of how to administer the test, and that it is quite user-friendly regardless of experience. On the other hand, it might also have been due to the two training occasions that had taken place prior to the data sampling, which in case may emphasize the importance of the preparations before using a test, whether for research purposes or clinical practice. In addition, the results also indicate that the Mini-BESTest consists of items that are difficult, yet important, to assess consistently in elderly with mild-to-moderate PD. Nevertheless, our results suggest that the Mini-BESTest is better suited to distinguish between individuals, rather than to achieve high agreement between test occasions in this population. Future studies, preferably on a more heterogeneous sample, need to investigate whether this may be due to the fluctuations of symptoms in PD [35,36] (something that might make this population difficult to assess reliably) or whether measures can be taken to increase the agreement of the Mini-BESTest (such as clearer instructions and increments).

### Limitations

This study has several limitations. First of all, our participants can be considered as a convenience sample, representing only elderly with mild-to-moderate PD who were interested in participating in a balance intervention, therefore our findings can be generalized only to this specific population. In addition, because the total Mini-BESTest scores in this study ranged from 15 to 25 points, we have investigated only this particular interval of the test. Although most training interventions in research [25,42,43], as well as in the clinic, tend to address individuals at the mild-to-moderate stage of the disease, clinical practice also includes treating individuals at more advanced stages of the disease. Moreover, our methodology of using raters with different experience was (although relevant with regard to clinical practice) rather strict- hence it is possible that the results would have been different if both raters had had similar experience. Furthermore, it is possible that the less-experienced test administrator experienced a learning effect during the course of data collection, a form of bias that also might have occurred with the participants as well as with the experienced test administrator at the test-retest assessments (that occurred 7 days after the initial assessments). However, we found no signs of this in our data.

### Clinical relevance

The importance for clinicians to be aware of the agreement of any clinical tool cannot be overestimated. Given the subjective aspects of any test, which include giving instructions and rating performance in general, it is important to know to what extent the outcome may depend upon a measurement error rather than on the actual test performance. This is even more critical in such a demanding task as evaluating an individual’s balance performance, particularly when considering its complex nature. Given the fall-prone nature of a population such as those with PD, where balance problems are all too frequent, this may be of particular value.

Some of the major challenges in obtaining reliable results from many balance tests lie in giving the patients clear and concise instructions while simultaneously ensuring that they will not fall and, at the same time, acknowledging their test performance with an adequate rating. This study has added important information regarding the reproducibility of the Mini-BESTest in elderly with mild-to-moderate PD assessed with a methodology assimilating clinical practice. As we investigated the SRD, these results can be used to evaluate individual treatment. Moreover, these results can also be applied to a group level, (by dividing the SRD found here, with the squared root of the number of participants investigated) [32]. Furthermore, this study also highlighted which subcomponents of the Mini-BESTest were the most difficult to assess consistently, indicating what aspects of the Mini-BESTest test might be beneficial to practice extra carefully prior to balance assessments in those with PD. Moreover, these results may also serve as an indicator of which subcomponents may need to be refined with regard to instructions to the patient as well as further clarifications of how the rating ought to be performed, in order to limit the subjective character of the test to as large an extent as possible. With such measures, we believe that there is great potential to enhance the clinical utility of such a promising test as the Mini-BESTest.

## Conclusions

The Mini-BESTest showed good inter-rater and test-retest reproducibility regarding reliability. However, regarding agreement, the measurement error was considered high, with postural responses being the subcomponent with the lowest agreement. This indicates that the Mini-BESTest is able to distinguish between individuals with mild to moderate PD; however, when used in clinical balance assessments, the large measurement error needs to be accounted for.
